# MEAN DIFFERENCE OF ZINC AND SELENIUM LEVELS AND TUBERCULOSIS OUTCOMES IN CHILDREN RECEIVING TREATMENT IN INDONESIA

**DOI:** 10.21010/Ajidv19i2S.9

**Published:** 2025-10-17

**Authors:** MASRUL Masrul, USMAN Elly, YANI Finny Fitry

**Affiliations:** 1 Department of Nutrition, Faculty of Medicine, Universitas Andalas, Padang, Indonesia; 2 Department of Pharmacology, Faculty of Medicine, Universitas Andalas, Padang, Indonesia; 3 Department of Child Health, Faculty of Medicine, Universitas Andalas, Padang, Indonesia

**Keywords:** Tuberculosis, Zinc, Selenium, Treatment, Outcome

## Abstract

**Background:**

Tuberculosis (TB) remains a significant health issue, particularly among children in TB-endemic regions. Micronutrient deficiencies, such as zinc and selenium, may influence TB treatment outcomes. However, the relationship between these micronutrients and TB outcomes in children is not well-established in Indonesia. This study aimed to investigate the mean difference of zinc and selenium levels on TB treatment outcomes in children receiving treatment in Padang, Indonesia.

**Materials and Methods:**

A cross-sectional study was conducted at the district level hospitals in Padang City, Indonesia, from April to October 2024. The study included children aged 1–14 years diagnosed with TB and receiving treatment. Serum zinc and selenium levels were measured using Chemiluminescent Immunoassay (ECLIA). The treatment outcomes were categorized as success (cured and completed treatment) or failure (death, treatment failure, or default).

**Results:**

Zinc levels were significantly lower in the failure group (71.61±8.18 ng/mL) compared to the success group (79.72±8.12 ng/mL) (P<0.05). Similarly, selenium levels were lower in the failure group (89.56±23.47 ng/mL) compared to the success group (115.09±17.86 ng/mL) (P<0.05).

**Conclusion:**

The study found a significant association between lower zinc and selenium levels and unsuccessful TB treatment outcomes in children. These findings suggest that addressing micronutrient deficiencies may play an important role in improving TB treatment outcomes in pediatric populations. Further research is needed to explore potential interventions to improve micronutrient status in TB patients.

## Introduction

Tuberculosis (TB) remains a significant source of morbidity and mortality among children. Out of the 9 million new TB infections annually, 11% occur in children (Koethe *et al.*, 2016; Sinha *et al.*, 2024). Malnutrition is one of the contributing factors and is also highly prevalent in children living in tuberculosis-endemic countries, contributing to 2.2 million deaths in children under the age of 5 globally (Dharmaraj *et al.*, 2025; Janssen *et al.*, 2025).

The interaction between TB and malnutrition is a major concern. TB can lead to weight loss, and malnutrition can predispose individuals to TB. On one hand, TB patients require more energy to maintain bodily functions due to an increased basal metabolic rate, which leads to weight loss (Su *et al.*, 2013; Grobler *et al.*, 2017). On the other hand, food intake can negatively affect TB patients due to reduced appetite and gastrointestinal disturbances, leading to nutritional deficiencies. Malnutrition further impairs immune function, as nutrient deficiencies alter the interaction between macrophages and T-lymphocytes (Ren *et al.*, 2019; Hao *et al.*, 2024). Moreover, although most individuals infected with TB will not show symptoms because their immune systems control the bacteria, individuals who are malnourished are more likely to develop active TB as the infection is no longer controlled by their immune systems ( Grobler *et al.*, 2017; Zheng *et al.*, 2024).

Nutritional assessment is crucial in the nutritional management of TB patients (Darnton-Hill *et al.*, 2022). Adequate nutritional intake is vital in combating TB. Insufficient protein and calorie intake can impair several essential host defense mechanisms needed to fight TB. Additionally, both vitamins and minerals play critical roles in immunity (Choi *et al.*, 2017). Deficiencies in one or more of these nutrients can compromise an individual’s ability to resist TB infection ( Botella *et al.*, 2011; Ramakrishnan *et al.*, 2012).

Micronutrient deficiencies are common in Indonesia, where the majority of the population relies on rice as a staple food, leading to low micronutrient intake, a condition that affects all age groups, including children. Zinc and selenium deficiencies in malnourished children are associated with impaired immunity and an increased risk of disease development (Ramakrishnan *et al.*, 2008; Stensland *et al.*, 2015). Reduced intake of micronutrients, particularly zinc and selenium, has been linked to immune dysfunction and a higher likelihood of latent TB progressing to active disease (Grobler *et al.*, 2017; Zheng *et al.*, 2024).

Zinc is utilized by the immune cells to destroy bacteria such as *Tuberculosis bacilli* or *Escherichia coli* (Amare *et al.*, 2015; Bacelo *et al.*, 2015). Lower zinc levels in the blood samples of TB patients have been reported in several previous studies. Many bacteria, such as *M. tuberculosis*, have transcriptional repressors responsive to metal-binding DNA that regulate the transcription process (Campa *et al.*, 2017; Tenforde *et al.*, 2017).

Selenium is a micronutrient essential for immunity against microorganisms. However, blood selenium levels are lower in patients with pulmonary tuberculosis and TB-HIV co-infection. Higher mortality rates among TB-infected patients have been linked to low blood selenium levels (Sepehri *et al.*, 2017; Nindrea *et al.*, 2020; Zheng *et al.*, 2024).

However, despite growing awareness of the link between malnutrition and TB, few studies have specifically quantified the association between serum zinc and selenium levels and treatment outcomes in pediatric TB patients, particularly in high-burden countries like Indonesia (Hendriyani *et al.*, 2020). Most existing literature has focused on adult populations or general nutritional status, without isolating the impact of these specific micronutrients on clinical outcomes in children. Furthermore, little is known about whether baseline levels of these micronutrients can serve as predictive markers for treatment response in pediatric TB cases (Nindrea *et al.*, 2019). This gap highlights the need for targeted research exploring these relationships in children, who represent a vulnerable and often overlooked group in TB research.

This study aims was to determine the mean difference of zinc and selenium levels and tuberculosis outcomes in children receiving treatment in Indonesia.

## Materials and Methods

### Study design and setting

This study utilized a cross-sectional research design. It was conducted at the district level hospitals in Padang City, Indonesia, from April to October 2024.

### Population and sample

The study population consisted of all children aged 1–14 years who were diagnosed with tuberculosis and were receiving TB treatment at the district level hospitals in Padang City. These children were identified based on data from the district level hospitals records. The sample included children aged 1–14 years with TB who were undergoing treatment, as documented at the district level hospitals. To determine the appropriate sample size for the study, a formula for two proportions was used. The sample size was calculated based on a 95% confidence level, with an α value of 0.05 for a two-tailed test. A previous study found that 92% of children with TB who had adequate zinc levels were cured (Mendes *et al.*, 2025), and the desired precision was set at 10%. This calculation resulted in a minimum sample size of 29 participants for the study.

### Sample criteria

The study included children aged 1–14 years who met the inclusion criteria and whose parents provided informed consent. The exclusion criteria were as follows: children who were diagnosed with tumors or other malignancies, those with severe infections such as measles, typhoid, HIV, or sepsis within the last two weeks, children with chronic kidney or liver diseases, and those receiving long-term medications, such as steroids or phenytoin.

### Sampling technique

The sampling technique involved simple random selection of children with TB undergoing treatment at the district level hospitals in Padang City. Children were selected based on their identities, which were recorded in the district level hospitals database.

### Study variables

The dependent variable in this study was the treatment outcome of tuberculosis in children. Treatment outcomes were classified as either success or failure. A successful outcome was defined as the child being cured and completing treatment, while failure was classified as death, treatment failure, default, or being transferred out (Cintron *et al.*, 2025). The independent variables were the zinc and selenium levels in the children’s serum. These were measured using the Chemiluminescent Immunoassay (ECLIA) technique, with the results expressed in ng/mL (Sinha *et al.*, 2025).

### Research procedure

Children who met the inclusion criteria were informed about the study, and written parental consent was obtained for blood sample collection. Once informed consent was received, blood samples were collected from each participant, and 5 milliliters of venous blood was drawn from each child.

### Ethical approval

This study received ethical approval from the Research Ethics Committee of Dr. M. Djamil General Hospital, Padang, Indonesia (No. DP.04.03/D.XVI.IV/246/2024).

### Data analysis

The data analysis process consisted of both univariate and bivariate analyses. For univariate analysis, categorical variables were presented as frequency tables and percentages, while numerical variables were summarized as measures of central tendency (mean ± standard deviation. For bivariate analysis, an Independent Samples T-test was used to examine the relationships between variables. A relationship was considered significant if the p-value was less than 0.05.

## Results

Characteristics of respondents (Table 1).

**Table 1:** Characteristics of respondents

Variable	Failure (n=18)	Success (n=11)
**Age of children (years), mean±SD**	5.17±2.94	6.82±3.37
**Sex, f(%)**		
Male	11 (73.3)	4 (26.7)
Female	7 (50.0)	7 (50.0)
**Age of mother (years), mean±SD**	29.83±6.87	28.09±7.09
**Mother’s education, f(%)**		
No school	1 (50.0)	1 (50.0)
Elementary school	4 (80.0)	1 (20.0)
Junior high school	8 (61.5)	5 (38.5)
Senior high school	4 (50.0)	4 (50.0)
University	1 (100.0)	0
**Mother’s occupation, f(%)**		
Housewife	12 (57.1)	9 (42.9)
Enterpreneur	6 (75.0)	2 (25.0)

Table 1 shows that children in the failure group had a lower average age (5.17 years) compared to the success group (6.82 years). The failure group had a higher proportion of males (73.3%) compared to the success group (26.7%), where the gender distribution was equal (50% male, 50% female). Mothers in the failure group were slightly older (29.83 years) than those in the success group (28.09 years). Regarding education, the majority of mothers in the failure group had completed elementary school (80%), while 50% of mothers in both groups had completed senior high school. One mother in the failure group had university education, but none in the success group did. For occupation, more mothers in the failure group were entrepreneurs (75%) compared to the success group (25%), while a higher proportion of mothers in both groups were housewives (57.1% in the failure group and 42.9% in the success group).

**Figure 1:**
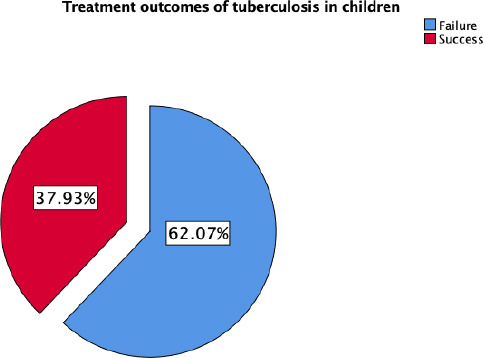
Treatment outcomes of tuberculosis in children

Treatment outcomes of tuberculosis in children (Figure 1).

Figure 1 showed that the treatment outcomes of tuberculosis among the respondents were primarily failure (62.07%) compared to success (37.93%).

Mean difference of zinc and selenium levels and tuberculosis outcomes in children receiving treatment in Indonesia (Table 2).

**Table 2:** Mean difference of zinc and selenium levels and tuberculosis outcomes in children receiving treatment in Indonesia

Variable	Failure (n=18) (Mean±SD)	Success (n=11) (Mean±SD)	P-value
Zinc (ng/mL)	71.61±8.18	79.72±8.12	0.015*
Selenium (ng/mL)	89.56±23.47	115.09±17.86	0.005*

P<0.05 considered significant

Table 2 showed that zinc levels were significantly lower in the failure group (71.61±8.18 ng/mL) compared to the success group (79.72±8.12 ng/mL) (P<0.05). Similarly, selenium levels were lower in the failure group (89.56±23.47 ng/mL) compared to the success group (115.09±17.86 ng/mL) (P<0.05).

## Discussion

This study found a significant association between lower zinc and selenium levels and unsuccessful tuberculosis (TB) treatment outcomes in children. The children in the failure group had notably lower zinc and selenium levels compared to the success group, with the differences in both micronutrients being statistically significant. These findings suggest that deficiencies in these key micronutrients may negatively affect TB treatment success in children, highlighting the importance of addressing these deficiencies to improve treatment outcomes (Janssen *et al.*, 2025; Yang *et al.*, 2025).

The role of micronutrients in immune function has been well documented in previous studies. Zinc, for example, is crucial for the proper functioning of immune cells and plays an important role in controlling the spread of *Mycobacterium tuberculosis*. Similarly, selenium is vital for immune responses and has been shown to influence the outcome of various infections, including TB (Grobler *et al.*, 2017; Zheng *et al.*, 2024). Previous research report supports our findings, showing that children with adequate zinc levels had better treatment outcomes (Sinha et al., 2024). Additionally, studies have shown that low selenium levels in TB patients are associated with poorer prognosis and higher mortality, reinforcing the significance of our results (Sepehri *et al.*, 2017; Zheng *et al.*, 2024).

Comparable findings have been reported in other high-burden regions. For example, studies from sub-Saharan Africa have similarly identified zinc and selenium deficiencies as risk factors for poor TB treatment outcomes, especially among children in low-income, food-insecure communities. However, some countries with robust nutritional programs such as Brazil and Thailand have reported better TB outcomes, partly due to systematic integration of micronutrient supplementation into TB care (Sepehri *et al.*, 2017; Nindrea *et al.*, 2024). Cultural dietary patterns also influence micronutrient intake; in Indonesia, a rice-dominated diet may contribute to lower trace mineral intake compared to regions with more diverse food sources (Nindrea *et al.*, 2024; Zheng *et al.*, 2024). Additionally, healthcare infrastructure, including the availability of diagnostic tools for micronutrient assessment and standardized nutritional interventions, varies widely between countries, potentially explaining differences in TB treatment success linked to nutritional status.

In practical terms, these findings suggest that micronutrient supplementation, specifically zinc and selenium, could be an effective strategy to improve TB treatment outcomes in children. Policymakers and healthcare providers in TB-endemic areas should consider incorporating micronutrient screening and supplementation into standard TB care protocols (Amare *et al.*, 2015; Bacelo *et al.*, 2015; Choi *et al.*, 2017). Public health initiatives aimed at educating communities about the importance of nutrition for preventing and treating TB could also be beneficial in reducing the burden of the disease (Botella *et al.*, 2011; Ramakrishnan *et al.*, 2012; Nindrea *et al.*, 2025).

While the study has provided valuable insights, it also has its limitations. The cross-sectional design of the study restricts the ability to establish a causal relationship between micronutrient deficiencies and TB treatment outcomes. Additionally, the relatively small sample size and the single-site nature of the study may limit the generalizability of the results. Despite these limitations, the study contributes significantly to the understanding of how micronutrient deficiencies may influence TB outcomes in children (Ren *et al.*, 2019; Hao *et al.*, 2024).

Looking ahead, future research should aim to conduct larger, multi-center cohort studies to further explore the causal link between micronutrient deficiencies and TB treatment failure. Randomized controlled trials assessing the impact of zinc and selenium supplementation on treatment outcomes would be valuable in determining the potential therapeutic benefits of these nutrients. Furthermore, longitudinal studies tracking changes in micronutrient levels over time could help identify whether early intervention with micronutrient supplementation leads to improved long-term outcomes for children with TB.

## Conclusion

The study identified a significant link between low levels of zinc and selenium and poor TB treatment outcomes in children. These results highlight the potential importance of correcting micronutrient deficiencies in enhancing TB treatment success in children. Additional studies are necessary to investigate possible interventions to improve micronutrient levels in TB patients. By identifying modifiable nutritional risk factors, this study lays the groundwork for future clinical and public health interventions aimed at improving treatment outcomes in pediatric TB patients.

### Conflict Of Interest

The authors declare that there is no conflict of interest associated with this study.

**List of Abbreviations:**
CI: Confidence interval;
ECLIA: Chemiluminescent Immunoassay;
HIV: Human Immunodeficiency Virus;
TB: Tuberculosis;
